# A Retrospective Study of Trifluridine/Tipiracil with Fruquintinib in Patients with Chemorefractory Metastatic Colorectal Cancer

**DOI:** 10.3390/jcm13010057

**Published:** 2023-12-21

**Authors:** Jiayun Zou, Yuanyuan Wang, Jiayu Xu, Jinna Li, Tianzhuo Wang, Ying Zhang, Yibo Bai

**Affiliations:** 1 Department of Oncology, Shengjing Hospital of China Medical University, Shenyang 110004, China; zoujy@sj-hospital.org (J.Z.); lijinna@sj-hospital.org (J.L.); 2 Department of Nephrology, Shengjing Hospital of China Medical University, Shenyang 110004, China; wangyy@sj-hospital.org; 3 Department of VIP In-Patient Ward, the First Hospital of China Medical University, Shenyang 110001, China; jiayuxu_cmu@163.com; 4 The First Clinical College, China Medical University, Shenyang 110122, China; tianzhuowang_cmu@163.com; 5 Department of Laboratory Medicine, Shengjing Hospital, China Medical University, Shenyang 110004, China; 6 Liaoning Clinical Research Center for Laboratory Medicine, Shenyang 110004, China

**Keywords:** trifluridine/tipiracil, fruquintinib, metastatic colorectal cancer, combination therapy

## Abstract

Introduction: Trifluridine/tipiracil (TAS-102) and fruquintinib are novel antitumor agents for patients with refractory metastatic colorectal cancer (mCRC). We conducted a retrospective study to explore the clinical efficacy and drug toxicities of combination therapy with TAS-102 and fruquintinib in real-life clinical practice. Methods: Between March 2021 and February 2023, patients at two different centers with mCRC who failed two or more lines of prior therapy and received TAS-102 in combination with fruquintinib were recruited. Results: In total, 32 mCRC patients were included in the analysis. The objective response rate (ORR) and the disease control rate (DCR) were 9.4% and 75%. The median progression-free survival (PFS) and overall survival (OS) were 6.3 (95% CI: 5.3–7.3) and 13.5 (95% CI: 9.5–17.5) months, respectively. Patients without liver metastasis or peritoneal metastasis obtained better median PFS (7.1 m vs. 5.6 m, *p* = 0.03 and 6.3 m vs. 3.4 m, *p* = 0.04), and OS (15.2 m vs. 10.4 m, *p* = 0.01 and 13.6 m vs. 7.1 m, *p* = 0.03), respectively. Other clinicopathological features, including age, tumor site, KRAS status, dosage of fruquintinib, and treatment line, did not affect the clinical efficacy of TAS-102 combined with fruquintinib. The most common grade three–four toxicities were neutropenia (46.9%), anemia (21.9%), diarrhea (15.6%), nausea (12.5%), and hand–foot syndrome rash (12.5%). Conclusions: Our results suggest that TAS-102 combined with fruquintinib has promising clinical efficacy and manageable safety for refractory mCRC patients in a real-world clinical setting. Further prospective trials are warranted to confirm our results.

## 1. Introduction

Colorectal cancer (CRC) is currently one of the most prevalent digestive system cancers. It is the third most common malignant neoplasm and was the second major reason for cancer-related mortality globally in 2020 [1]. About 15–30% of patients with CRC exhibit metastasis at presentation, while approximately 20–50% with initially localized disease may advance to metastasis [2]. The anticipated 5-year survival rate for unresectable metastatic colorectal cancer (mCRC) is less than 5% [3]. With the emerging era of individualized precision treatment for mCRC, treatment options need to be tailored based on molecular profiling (RAS/RAF/MSI/HER2/NTRK) [4,5]. Despite significant improvements in survival outcomes for mCRC due to personalized comprehensive treatment, including advancing chemotherapies, targeted drugs, and immune checkpoint inhibitors, some mCRC patients with oncogenic drivers are not druggable, making precision treatment unfeasible for them, and the prognosis of mCRC declines drastically when in refractory status [2]. Thus, effective and less toxic treatments irrespective of molecular markers for refractory mCRC are urgently needed to further improve disease outcomes. At present, the recommended treatment for refractory mCRC comprises regorafenib, trifluridine/tipiracil (FTD/TPI and TAS-102), and fruquintinib, as stated in the guidelines of the Chinese Society of Clinical Oncology (CSCO) and the European Society for Medical Oncology (ESMO) [2,6]. Nonetheless, the optimal utilization of these agents, including their combination or sequence, remains uncertain.

TAS-102 is a new oral drug comprising trifluridine, a nucleoside analog based on thymidine, and tipiracil hydrochloride, a thymidine phosphorylase antagonist that enhances the bioavailability of the drug [7]. Two phase III trials have shown that TAS-102 provides clinical benefits in refractory mCRC patients, whereas the differences in survival were found to be numerically modest [8,9]. In order to further improve antitumor activity, different combined therapies are in the exploratory stage. Among them, TAS-102 plus bevacizumab is the most promising regimen. Two phase II studies evaluated TAS-102 combined with bevacizumab in chemorefractory mCRC and confirmed its encouraging efficacy with a desirable safety profile [10,11]. Another phase III study (SUNLIGHT) also compared TAS-102 plus bevacizumab with TAS-102 alone in refractory patients, indicating a substantial increase in PFS and OS with tolerable compliance [12].

However, most mCRC patients receive long-term treatment with bevacizumab, and resistance to bevacizumab frequently occurs during the course of treatment [13,14]. Fruquintinib is a highly selective vascular endothelial growth factor receptor (VEGFR) inhibitor targeting VEGFR1, 2, and 3. Clinical research has demonstrated that patients with bevacizumab-resistant mCRC can acquire benefits from fruquintinib [15,16]. A study involving colorectal cancer xenografts found that phosphorylated FTD levels were increased in tumors by combining TAS-102 and an angiogenesis inhibitor, resulting in increased antitumor activity [17]. In addition, preclinical models illustrated that FTD/TPI and fruquintinib led to a notable decrease in the microvessel area and exhibited remarkable superiority over each monotherapy studied in mice bearing subcutaneous colorectal tumor xenografts [18]. Hence, their unique mechanisms suggest that they may be complementary when received in combination. In the same scenario, a phase II study is presently being conducted to assess the effectiveness and safety of TAS-102 combined with fruquintinib (NCT05004831).

Until now, evidence supporting the role of TAS-102 plus fruquintinib in a clinical practice setting has been limited. Thus, we performed an observational study to analyze the clinical effectiveness and drug toxicities of TAS-102 combined with fruquintinib for chemorefractory mCRC patients in a multicenter real-world clinical setting.

## 2. Materials and Methods

### 2.1. Patients

This retrospective analysis included patients with mCRC who failed at least two lines of standard treatment and received TAS-102 in combination with fruquintinib as later-line treatment in two different centers (the First Hospital of China Medical University and Shengjing Hospital of China Medical University) from March 2021 to February 2023. The eligibility criteria were as follows: patients over 18 years old with histologically verified adenocarcinoma and at least one detectable lesion based on the Response Evaluation Criteria in Solid Tumors (RECIST) version 1.1; a history of regimens using fluoropyrimidine, irinotecan, oxaliplatin, bevacizumab, or cetuximab for patients with RAS wild-type mCRC; and an Eastern Cooperative Oncology Group performance status (ECOG PS) of 0 to 2. The exclusion criteria were prior treatment with TAS-102 or fruquintinib, combined treatment of less than two cycles, or patients lost at follow-up.

### 2.2. Treatments

Fruquintinib was administered orally in two different doses of 4 mg or 3 mg regularly on days 1–21 with a 7-day break. TAS-102 was taken at 35 mg/m^2^ orally twice daily on days 1–5 and 8–12 of a 28-day cycle. Physicians could make adjustments to the dosage based on patient complications and adverse effects. Therapy was continued until illness progression, death, intolerable toxicities, a patient’s choice, or a physician’s decision to terminate therapy.

### 2.3. Efficacy and Safety Assessments

The evaluated endpoints included the objective response rate (ORR), disease control rate (DCR), progression-free survival (PFS), overall survival (OS), and adverse events (AEs). Computed tomography was evaluated for the treatment response according to RECIST v1.1 after every 2 cycles of combined therapy, including complete response (CR), partial response (PR), stable disease (SD), and progressive disease (PD). ORR refers to the proportion of patients with CR or PR, and DCR refers to the proportion of patients with CR, PR, and SD. AEs were noted and ranked based on the Common Terminology Criteria for Adverse Events (version 5.0) [19].

### 2.4. Statistical Analysis

Continuous variables are presented as the median with a range of values, and categorical data are reported as frequencies and percentages. The chi-square test was used to determine differences between the categorical data. Follow-up time was defined as the span from the initiation of treatment to the final follow-up date for censored cases. The follow-up time ended on 30 June 2023. Survival curves were obtained through the use of the Kaplan–Meier method and compared using the log-rank test. The Cox regression model was utilized to determine hazard ratios (HRs) and corresponding 95% confidence intervals (CIs). Statistical analyses were conducted with SPSS 29. *p* < 0.05 was deemed to be statistically significant.

## 3. Results

### 3.1. Patient and Treatment Characteristics

We recorded 35 patients who received fruquintinib and TAS-102 between 1 March 2021 and 30 February 2023 at two different institutions. Three patients did not complete the first assessment due to AEs or other personal reasons and were not included in subsequent analyses. Eleven patients received fruquintinib at a dose of 3 mg, while the others received the drug at a dose of 4 mg. The median age of our patients was 64 years (range 39–78 years). The patients’ baseline characteristics are presented in Table 1. In this cohort, 40.6% of the patients were female, and 21.9% had an ECOG PS of 0 at screening. Left-sided tumors accounted for 65.6% of the tumors found in the patients, 53.1% had synchronous presentation, 56% were RAS mutant, 6% were BRAF mutant, and 40.6% of the patients had three or more metastatic sites. The liver and lungs were the most frequent metastatic locations, contributing to 68.8% and 62.5% of the total, respectively. Moreover, 59.4% of the patients had lymph node metastases, and 25% had peritoneum carcinomatosis. All of the patients were microsatellite stable (MSS) and had received prior standard therapy. Thus, 22 (68.8%) patients received TAS-102 combined with fruquintinib as a third-line treatment, and 10 (31.2%) patients received this combination as a fourth- or latter-line treatment.

### 3.2. Efficacy

No patient had a CR, and three patients were assessed as PR in the entire group (Table 2). The ORR and DCR were found to be 9.4% and 75%, respectively. The patients who achieved PR were on TAS-102 plus fruquintinib at a dose of 4 mg. There was no statistical significance in terms of efficacy with regard to all clinicopathological characteristics.

**Figure 1:**
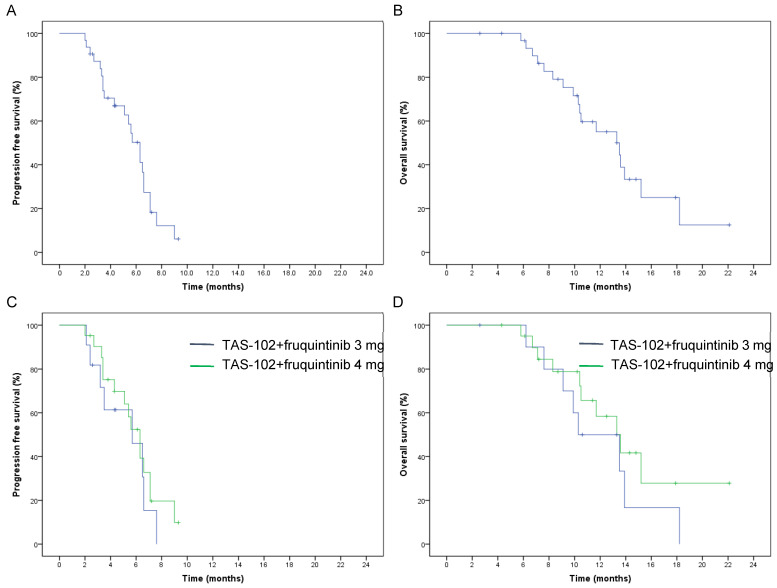
Kaplan–Meier analysis of (**A**) progression-free survival and (**B**) overall survival in the whole population. Kaplan–Meier analysis of (**C**) progression-free survival and (**D**) overall survival in different doses of fruquintinib.

**Figure 2:**
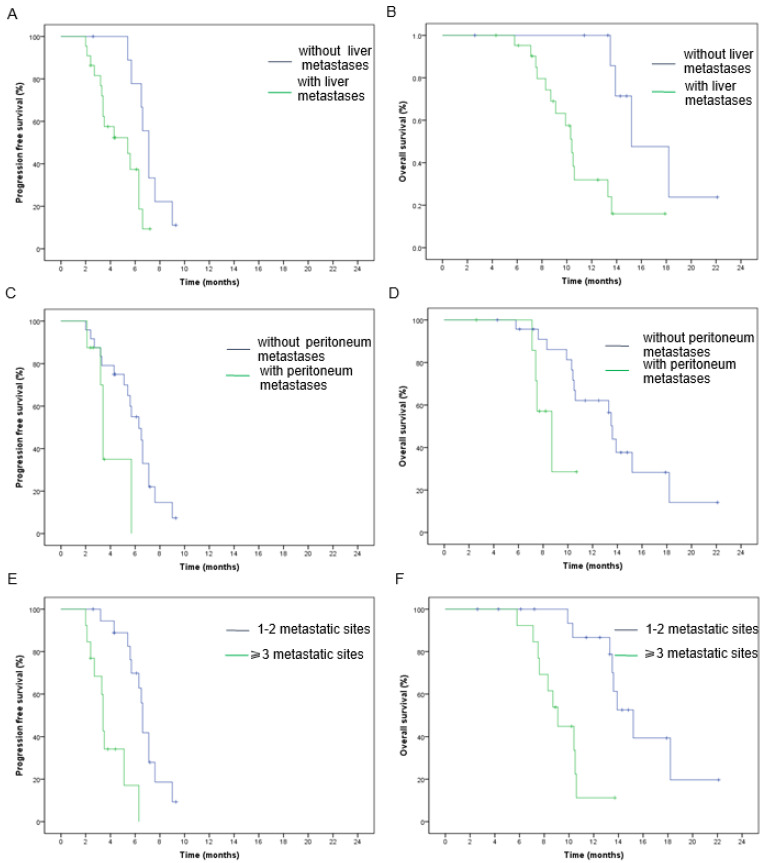
Kaplan–Meier analysis of (**A**) progression-free survival and (**B**) overall survival in patients with and without liver metastases. Kaplan–Meier analysis of (**C**) progression-free survival and (**D**) overall survival in patients with and without peritoneum metastases. Kaplan–Meier analysis of (**E**) progression-free survival and (**F**) overall survival in patients with 1–2 and ≥3 metastatic sites.

**Figure 3:**
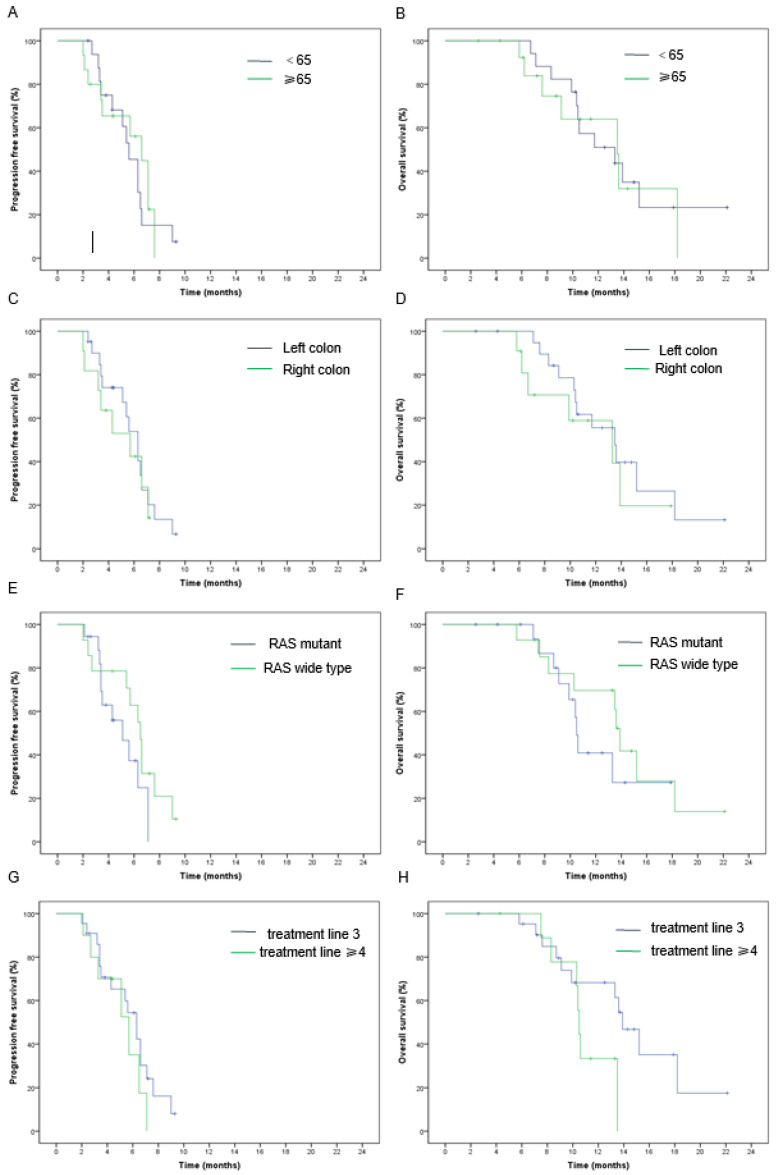
Kaplan–Meier analysis of (**A**) progression-free survival and (**B**) overall survival in <65 and ≥65 population. Kaplan–Meier analysis of (**C**) progression-free survival and (**D**) overall survival in different tumor sites. Kaplan–Meier analysis of (**E**) progression-free survival and (**F**) overall survival in different RAS statuses. Kaplan–Meier analysis of (**G**) progression-free survival and (**H**) overall survival in different treatment lines.

The median PFS and OS in the entire group were 6.3 months (95% CI: 5.3–7.3) and 13.5 months (95% CI: 10–17), respectively (Figure 1A,B). Patients without liver metastases had higher PFS (7.1 vs. 5.4 m, *p* = 0.01) and OS (15.2 vs. 10.4 m, *p* = 0.003) values than those with liver metastases (Figure 2A,B). Similarly, patients without peritoneal metastases had superior PFS (6.3 m vs. 3.4 m, *p* = 0.04) and OS (13.6 m vs. 8.7 m, *p* = 0.03) values compared to patients with peritoneal metastases (Figure 2C,D). The number of metastatic sites is a prognostic factor in survival outcomes. The PFS (6.6 m vs. 3.4 m, *p* = 0.000) and OS (15.2 m vs. 9.1 m, *p* = 0.000) in patients who had one–two metastatic sites were higher than in patients with three or more metastatic sites (Figure 2E,F). However, none of the other clinicopathological parameters showed statistical differences in terms of survival benefits (Figure 3).

### 3.3. Safety

The AEs that occurred during treatment are summarized in Table 3. Hematological toxicity was the predominant adverse event. The most frequent Grade 3–4 toxicities also concerned hematological toxicity, including neutropenia (46.9%), anemia (21.9%), and decreased platelet count (6.3%). The common nonhematological AEs were nausea (53.1%), fatigue (50%), diarrhea (43.8%), hypertension (46.9%), proteinuria (31.3%), and oral mucositis (31.3%). The majority of these AEs were Grade 1–2 in severity. However, the incidence of Grade 3–4 diarrhea was 15.6%, followed by nausea (12.5%) and hand–foot syndrome rash (12.5%). These AEs can result in severe suffering and necessitate dose reduction and therapy cessation. Dose reduction occurred in 12 (37.5%) patients (Table 4). For TAS-102, eight (25%) patients received one dose reduction (from 35 mg/m^2^ to 30 mg/m^2^), and five (15.6%) patients received two dose reductions (from 35 mg/m^2^ to 25 mg/m^2^). As for fruquintinib, six (28.6%) patients received one dose reduction (from 4 mg to 3 mg). Overall, nine (28.1%) patients required treatment interruptions due to AEs, and cessation occurred in five (15.6%) patients. There were no treatment-related fatalities, and most AEs related to both drugs were manageable.

## 4. Discussion

This is the first study to evaluate the activity and safety of the combination of TAS-102 and fruquintinib for refractory mCRC patients in a real-life setting. Our current study indicates that combination therapy led to better outcomes in terms of DCR, ORR, PFS, and OS than monotherapy with TAS102 or fruquintinib based on previous prospective clinical trials [8,9,15]. Moreover, the efficacy of TAS-102 plus fruquintinib in our study seemed to have an advantage over TAS-102 plus bevacizumab, according to earlier clinical data [10].

For mCRC patients who are refractory to front-line conventional treatments, drug resistance always occurs, limiting the efficacy of third-line treatment. Recently, the advent of two novel agents, TAS-102 and fruquintinib, has shown survival benefits, and they are currently the recommended third-line therapies. TAS-102 has shown significant antitumor activity in vitro and in xenograft models and can reverse resistance induced by thymidylate synthase (TS) overexpression in cancer cells refractory to 5-fluorouracil (5-FU) [20,21]. In order to attain superior clinical efficacy, several preclinical studies have demonstrated encouraging synergistic effects between TAS-102 and other chemotherapeutic agents, such as oxaliplatin [22], irinotecan [23], bevacizumab, cetuximab, and panitumumab [17]. Among these combinations, the antitumor therapy option of TAS-102 with bevacizumab appears to be the most effective [17]. TAS-102 selectively targets thymidine phosphorylase (TP), which functions synergistically with anti-VEGF-targeted treatment to prevent tumor angiogenesis [24]. Moreover, the combination of TAS-102 and bevacizumab leads to elevated levels of trifluorothymidine and its cytotoxic phosphorylated metabolites within the tumor in comparison to TAS-102 monotherapy, which implies a potential mechanism for increased efficacy through this combination [17]. Clinical trials involving TAS-102 plus bevacizumab have consistently demonstrated an advantage, both in terms of median PFS and OS of around 2–3 months compared with TAS-102 alone [10,11,12].

However, bevacizumab only targets the VEGF-A ligand, and the bypass is activated. Resistance to bevacizumab is mostly associated with a decrease in VEGF-A and an increase in PDGF, VEGF-C, and VEGF-D levels after long-term therapy [13,25]. Hence, there is a need for an agent that can target multiple signaling pathways. Regorafenib and fruquintinib are small-molecule inhibitors able to hinder multiple kinases, including VEGFR, FGFR, and PDGFR [26,27]. Studies using colorectal cancer xenograft models have found that fruquintinib combined with FTD/TPI can enhance antitumor effects [18]. In addition, compared with regorafenib, fruquintinib has a narrower spectrum of targets and a lower incidence of AEs, which might enhance therapeutic efficacy through increased drug exposure at the maximum tolerated dose [26,27]. Moreover, fruquintinib is a cost-effective choice for pretreated mCRC patients compared to oral regorafenib [28]. We therefore believe that the combined administration of TAS-102 and fruquintinib might be a valuable treatment regimen for refractory mCRC.

In this retrospective analysis, we present data from 32 refractory mCRC patients receiving TAS-102 plus fruquintinib at two different institutions. The results were encouraging, and although no patient achieved CR, three patients achieved PR, resulting in an ORR of 9.4% and a DCR of 75%. With regard to efficacy, the median PFS and OS were 6.3 and 13.5 months, respectively. SUNLIGHT, a phase III clinical study evaluating TAS-102 combined with bevacizumab, achieved median PFS and OS of 5.6 months and 10.8 months, respectively [12]. Our results showed greater improvements in survival with the administration of TAS-102 plus fruquintinib compared with the results of the SUNLIGHT study.

The liver is one of the most common sites of metastases in colorectal cancer. Nearly one-half of patients will be diagnosed with liver metastasis during the progression of the disease, which is hazardous to survival [29]. The possibility of peritoneal carcinomatosis in CRC patients is 25% and is insensitive to modern chemotherapy regimens [29,30]. Our analysis also revealed poorer PFS and OS in patients with either hepatic or peritoneal metastases.

The RECOURSE and TERRA studies have demonstrated that TAS-102 provides superior survival benefits in senior patients over 65 years of age [8,9]. The FRESCO and FRESCO-2 studies also revealed no difference in the efficacy or frequency of adverse events with fruquintinib across age groups [15,16]. The Q-TWiST analysis involved an evaluation of the survival and quality of life of mCRC patients administered fruquintinib, which showed clinically significant benefits [31]. Our analysis pertained to the efficacy and safety of TAS-102 combined with fruquintinib in elderly patients. Despite the lack of statistical significance on account of the small sample size, greater efficacy and better survival were seen in senior patients over 65 years of age. In addition, our study shows that higher ORR is linked to left-sided tumors and RAS wild-type tumors, similar to the results described earlier by Kuboki et al., although in a different combination treatment [10].

In general, sustained treatment and enhanced adherence are crucial for improving therapeutic efficacy. Liu et al. conducted a study involving a questionnaire assessing patient preference for oral versus intravenous chemotherapy, which showed a clear preference for oral drugs with the condition of not sacrificing efficacy [32]. TAS-102 and fruquintinib are both oral drugs, and this combination is well tolerated, which makes it a suitable option for long-term treatment, especially during the COVID-19 epidemic, minimizing hospitalization. In terms of whether administration order can affect antitumor activity, preclinical studies show that sequential treatment with FTD followed by regorafenib has superior effectiveness in colorectal cancer cells compared to simultaneous treatment or regorafenib followed by FTD exposure [33]. Regorafenib can reduce FTD-induced TS expression, and this may enhance antitumor efficacy when cells are post-treated with regorafenib. In addition, post-treated regorafenib may not affect the insertion of FTD into DNA [33]. Both fruquintinib and regorafenib are oral multikinase inhibitors with antiangiogenic properties and are alternative treatment options for heavily pretreated mCRC. We therefore believe that the best administration sequence, whether simultaneously or sequentially, needs to be further investigated.

With regard to the safety profile of this treatment, the combination of TAS-102 and fruquintinib exhibited a tolerable toxicity profile in a clinical practice setting. There was a greater incidence of hematological toxicity (neutropenia and anemia) with TAS102 plus fruquintinib than with TAS102 monotherapy from prior reports of treatment, especially in patients who were administered fruquintinib at a dose of 4 mg. These adverse events mainly caused dose adjustments and treatment discontinuation. Although the frequency of proteinuria and hypertension did not increase in this analysis, we should closely monitor patients receiving fruquintinib in this combination setting because most mCRC patients have been exposed to angiogenesis inhibitors as front-line treatment. Other major toxicities are fatigue, nausea, and diarrhea, and most of these side effects are Grade 1–2 in severity, which indicates that the combined treatment did not increase the frequency of these side effects. In brief, the decision reached on the specific drug dosage is important for patients, and many factors should be considered, including ECOG PS, tumor burden, patient comorbidities, metastatic sites or numbers, mutational status, and toxicity profile.

In the future, we need to identify clinical and molecular biomarkers to predict clinical benefits. Recent studies have revealed that FTD incorporation into DNA differs depending on disparities in the substrate specificities of TK1 and deoxyUTPase (DUT). Hence, TK1 and DUT may serve as potential biomarkers capable of identifying patients who will benefit from TAS-102 [34]. In addition, serum LDH, hERG1, HIF-2, and circulating angiopoietin-2 levels have been found to be novel biomarkers associated with responses to antiangiogenic drugs [35,36,37]. A post hoc analysis of the FRESCO trial demonstrated a link between HFS response and survival advantages gained from fruquintinib administration [38]. These predictive biomarkers need further validation and may help to evaluate this combination therapy as an appropriate treatment for mCRC refractory to standard therapies. The next steps in the clinical management of mCRC patients will be to identify CRC subtypes, such as colitis-associated colon cancer, and apply system biology to integrate tumor gene alterations, microenvironment genes, protein expression, intestinal bacteria species, and their dynamic changes during the disease course for genuinely individualized precision medicine [39].

This study has some potential limitations, and one should not draw broader conclusions from these data. Firstly, the study was a retrospective analysis with a small cohort of patients. Meanwhile, all the patients involved in our analysis were Chinese patients treated at two institutions without ethnic differences, which may constrain the power of the study. Secondly, the dose of fruquintinib, 3 mg or 4 mg, was determined by doctors based on the patients’ ECOG PS, complications, and the number of prior regimen lines, which may represent a major bias in our study. Thirdly, an assessment of the patients’ quality of life, a significant outcome in the salvage-line setting, was not performed. Lastly, there is a lack of system biology analysis and preclinical basic research available to explore the underlying mechanisms. Given this, our study initially concluded that TAS-102 plus fruquintinib seems more effective and safer than current treatment options. At present, there have been no large-scale prospective randomized controlled trials targeting this combination therapy. Therefore, despite the inherent selection bias present in our retrospective analysis, it still has clinical value.

## 5. Conclusions

Our retrospective study results suggest that TAS-102 combined with fruquintinib has promising clinical efficacy and manageable safety for refractory mCRC patients in a real-world clinical setting. These conclusions have therapeutic significance and open new avenues for the treatment of refractory mCRC. In the future, the detection of potential predictive biomarkers is warranted to investigate the optimal patient benefits of this combination therapy, and further prospective randomized controlled trials are necessary to confirm our findings.

## Figures and Tables

**Table 1:** Patient characteristics.

Characteristic	Total (*n* = 32) *n* (%)	TAS-102 Plus Fruquintinib 4 mg (*n* = 21) *n* (%)	TAS-102 Plus Fruquintinib3 mg (*n* = 11) *n* (%)	*p*
Age (median, range)	64 (39–78)	58 (39–74)	67 (46–78)	
Sex				1
Male	19 (59.4)	12 (57.1)	7 (63.6)	
Female	13 (40.6)	9 (42.9)	4 (36.4)	
ECOG				0.02
0	7 (21.9)	7 (33.3)	0 (0)	
1	20 (62.5)	13 (61.9)	7 (63.6)	
2	5 (15.6)	1 (4.8)	4 (36.4)	
Primary location				1
Right colon	11 (34.4)	7 (33.3)	4 (36.4)	
Left colon	21 (65.6)	14 (66.7)	7 (63.6)	
Number of metastatic sites				1
1–2	19 (59.4)	12 (57.1)	7 (63.6)	
≥3	13 (40.6)	9 (42.9)	4 (36.4)	
Metastatic sites				0.45
Liver	22 (68.8)	16 (76.2)	6 (54.5)	
Lung	20 (62.5)	10 (47.6)	10 (90.9)	
Peritoneum	8 (25)	5 (23.8)	3 (27.3)	
Lymph node	19 (59.4)	11 (52.4)	8 (72.7)	
Others	2 (6.25)	2 (9.5)	0 (0)	
RAS status				0.47
Wild-type	14 (44)	8 (38.1)	6 (54.5)	
Mutant	18 (56)	13 (61.9)	5 (45.5)	
BRAF status				1
Wild-type	30 (93.8)	20 (95.2)	10 (90.9)	
Mutant	2 (6.2)	1 (4.8)	1 (9.1)	
MMR status				
MSS	32 (100)	21 (100)	11 (100)	
Unknown	0 (0)	0 (0)	0 (0)	
Treatment line				0.06
3	22 (68.8)	17 (81)	5 (45.5)	
≥4	10 (31.2)	4 (19)	6 (54.5)	
Previous treatment agents				0.99
Fluorouracil	32 (100)	21 (100)	11 (100)	
Oxaliplatin	32 (100)	21 (100)	11 (100)	
Irinotecan	30 (93.8)	20 (95.2)	10 (90.9)	
Raltitrexed	3 (9.4)	2 (9.5)	1 (9.1)	
Bevacizumab	29 (90.6)	20 (95.2)	9 (81.8)	
Cetuximab	8 (25)	5 (23.8)	3 (27.3)	
Regorafenib	4 (12.5)	2 (9.5)	2 (18.2)	

ECOG, Eastern Cooperative Oncology Group; MMR, mismatch repair; MSS, microsatellite stable; TAS-102, trifluridine/tipiracil.

**Table 2:** Overall response.

	ORR	*p*	DCR	*p*	Median PFS (95% CI)	*p*	Median OS (95% CI)	*p*
Total	9.4%		75%		6.3 (5.3–7.3)		13.5 (9.5–17.5)	
Treatment program		0.53		0.40		0.40		0.12
TAS-102 + fruquintinib 3 mg	0		63.6%		5.7 (2.3–9.1)		10.3 (9.2–11.4)	
TAS-102 + fruquintinib 4 mg	14.3%		81%		6.3 (5.2–7.4)		13.6 (11.8–15.4)	
Age		0.59		0.69		0.85		0.87
<65	5.9%		70.6%		5.4 (4.9–5.9)		13.3 (8.2–13.4)	
≥65	13.3%		80%		6.5 (5.0–8.0)		13.5 (10.2–16.8)	
Tumor site		1		0.40		0.61		0.46
Left	9.5%		81%		6.3 (5.2–7.4)		13.5 (8.4–18.6)	
Right	9.1%		63.6%		5.7 (2.4–9.0)		13.3 (6.6–20)	
RAS status		0.57		0.70		0.17		0.86
Wild	14.3%		71.4%		6.5 (5.5–7.5)		13.6 (8.5–18.7)	
Mutant	5.6%		77.8%		5.4 (3.7–7.1)		10.6 (6.9–14.3)	
Metastatic site		0.22		1		0.03		0.01
Liver	4.5%		72.7%		5.6 (3.2–8.0)		10.4 (9.5–11.3)	
Without liver	20%		80%		7.1 (6.3–7.9)		15.2 (10.9–19.5)	
Metastatic site		0.56		0.38		0.04		0.03
Peritoneum	0		62.5%		3.4 (3.0–3.8)		7.1 (4.1–10.1)	
Without peritoneum	12%		79.2%		6.3 (4.7–7.9)		13.6 (12.9–14.3)	
Number of metastatic sites		1		0.22		0.000		0.000
1–2	10.5%		84.2%		6.6 (6.4–6.8)		15.2 (13–17.4)	
≥3	7.7%		61.5%		3.4 (3.2–3.6)		9.1 (6.6–11.6)	
Treatment line		0.53		0.68		0.34		0.22
3	13.6%		77.3%		6.3 (5.1–7.5)		13.6 (11–16.2)	
≥4	0		70%		5.7 (3.0–8.4)		10.5 (10.0–11.1)	

DCR, disease control rate; OS, overall survival; PFS, progression-free survival; TAS-102, trifluridine/tipiracil.

**Table 3:** Adverse events.

Adverse Event	All Grades	≥Grade 3
Neutropenia	26 (81.3)	15 (46.9)
Anemia	17 (53.1)	7 (21.9)
Decreased platelet	13 (40.6)	2 (6.3)
Elevated ALT/AST	7 (21.9)	0
Elevated bilirubin	4 (12.5)	1 (3.1)
Fatigue	16 (50)	2 (6.3)
Nausea	17 (53.1)	4 (12.5)
Diarrhea	14 (43.8)	5 (15.6)
Oral mucositis	10 (31.3)	2 (6.3)
Hypertension	15 (46.9)	3 (9.4)
Proteinuria	10 (31.3)	2 (6.3)
HFS	9 (28.1)	4 (12.5)
dysphonia	6 (18.8)	3 (9.4)
Abdominal pain	3 (9.4)	0
Constipation	4 (12.5)	0
Hemorrhage	5 (15.6)	0

HFS, hand-and-foot syndrome reaction.

**Table 4:** Dose adjustment.

Number of the Patients with Dose Reduction	TAS-102	Fruquintinib
One-level dose reduction	8 (25%)	6 (28.6%)
Two-level dose reduction	5 (15.6%)	0 (0%)
Number of patients with treatment interruption	9 (28.1%)	9 (28.1%)
Number of patients with treatment discontinuation	5 (15.6%)	2 (6.3%)

## Data Availability

Data are available upon request.
